# High-Accuracy Readout Electronics for Piezoresistive Tactile Sensors

**DOI:** 10.3390/s17112513

**Published:** 2017-11-01

**Authors:** José A. Hidalgo-López, Óscar Oballe-Peinado, Julián Castellanos-Ramos, José A. Sánchez-Durán, Raquel Fernández-Ramos, Fernando Vidal-Verdú

**Affiliations:** 1 Departamento de Electrónica, Universidad de Málaga, Andalucía Tech, Campus de Teatinos, Málaga 29071, Spain; oballe@uma.es (Ó.O.-P.); jcramos@uma.es (J.C.-R.); jsd@uma.es (J.A.S.-D.); rfernandezr@uma.es (R.F.-R.); fvidal@uma.es (F.V.-V.); 2 Instituto de Investigación Biomédica de Málaga (IBIMA), Málaga 29010, Spain

**Keywords:** resistive sensor arrays, parallel analog data acquisition, piezoresistive tactile sensors

## Abstract

The typical layout in a piezoresistive tactile sensor arranges individual sensors to form an array with *M* rows and *N* columns. While this layout reduces the wiring involved, it does not allow the values of the sensor resistors to be measured individually due to the appearance of crosstalk caused by the nonidealities of the array reading circuits. In this paper, two reading methods that minimize errors resulting from this phenomenon are assessed by designing an electronic system for array reading, and the results are compared to those obtained using the traditional method, obviating the nonidealities of the reading circuit. The different models were compared by testing the system with an array of discrete resistors. The system was later connected to a tactile sensor with 8 × 7 taxels.

## 1. Introduction

Resistive sensors are used in a large number of applications to measure physical variables. In some applications, these sensors are used individually, while in other cases, a large number are required in order to measure physical magnitude. These groups of sensors are used in multiple applications: light-dependent applications [1,2], gas detectors [3,4,5], temperature sensors [6,7], thermal anemometry [8] and tactile sensors or artificial electronic skins [9,10,11,12,13,14,15,16,17,18,19]. The number of individual sensors tends to be high, especially in the case of tactile sensors or artificial electronic skins.

**Figure 1:**
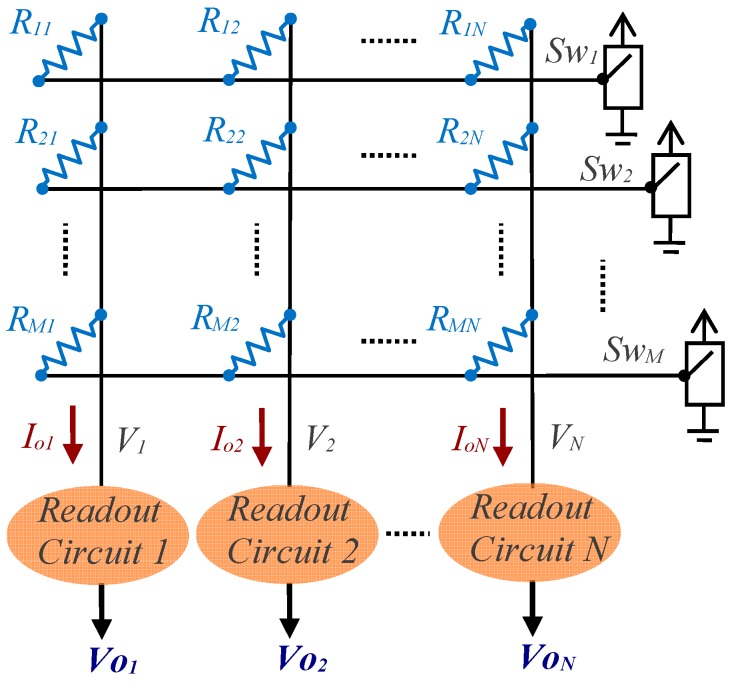
Basic structure of electronics for reading a resistive sensor array.

A naïve approach to solving the problem of reading the electronics of a large number of sensors simultaneously is to design a reading circuit for each of them. However, this is very costly in terms of both hardware and area. A widely used solution to avoid these drawbacks consists of organising the sensors in the form of an array with *M* rows and *N* columns, Figure 1, such that each sensor (represented by a resistor) is connected to a row wire and a column wire. There are also sensors that have this structure as a result of their very manufacture; this is especially prevalent in tactile sensors [15,19].

The reading procedure for the circuit shown in Figure 1 consists of using the *Sw_i_* switch to select row *i* at a positive voltage value, while the other switches are connected to ground (these switches can be constructed using simple digital buffers). The result is that the readout circuits maintain zero voltage in the vertical wires, the current will enter these circuits through a single resistor per column, and it is possible, to some degree, to evaluate the value of these resistors. This is known as the Zero Potential Method. The time required to evaluate the array resistor values is *M*·*t_r_*, where *t_r_* is the time needed to evaluate the value in a single sensor.

The Zero Potential Method also allows the reading circuit to be constructed using both a single readout circuit [20,21,22,23,24,25] and a series of additional multiplexers to control the row and column wires. However, if the number of sensors in these circuits is high, given that the time required to read all the sensors is *M*·*N*·*t_r_*, the time needed may be too great to evaluate important characteristics in tactile arrays such as grip or slippage.

**Figure 2:**
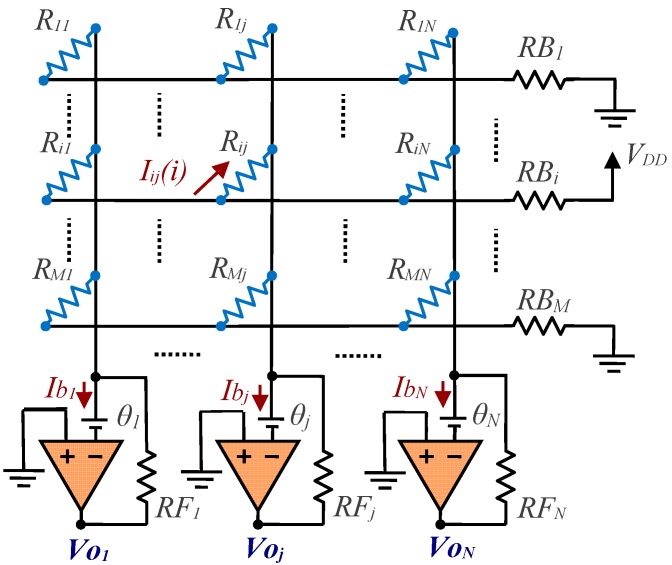
Implementation of the resistor array reading circuit with operational amplifiers (OAs).

However, the circuit shown in Figure 1 (as well as the solutions with a single readout circuit) presents practical problems in ensuring that the current that enters each readout circuit is solely dependent on the value of a single resistor in each column. Firstly, we have the output resistors for each switch; and secondly, the nonidealities of the readout circuits themselves. This can be analysed in a practical implementation of the circuit of Figure 1, as shown in Figure 2, where the row *i* switch has been selected. In this circuit, each switch has been replaced by its output resistor, *RB_i_* (which can have a different value for each switch status), and the readout circuit has been constructed with an operational amplifier (OA) with negative feedback and a resistor. The main error sources in this type of readout circuit are the offset voltage, *θ_j_*, (shown in Figure 2), the bias currents, *Ib_j_*, and the finite gain, *A_j_*, of each OA.

Due to *RB_i_*, *θ_j_*, *Ib_j_*, and *A_j_*, the current that circulates through the wire of each column does not depend solely on the value of the resistor in a single sensor, but is also influenced by the values of the other array resistors. This is known as the crosstalk effect [6,20,26,27,28].

If we ignore these sources of error and assume *RB_i_* = 0, *θ_j_* = 0, *Ib_j_* = 0, and, *A_j_* → ∞, in Figure 2 we can obtain *R_ij_* from the value of *Vo_j_*:

(1) $$R_{ij}=-\frac{V_{DD}}{Vo_{j}}RF_{j}$$

However, due to these nonideal parameters, the result of using Equation (1) shows a certain degree of error, such as in a previous study [23], with errors of 30% for a 4 × 4 array with resistors in a range between 100 and 1000 Ω, using buffers with an internal resistance of 10 Ω to address the rows. Errors of over 40% can occur, depending on the type of resistor to be measured, and even using two readout circuits to minimise errors [29]. The occurrence of phantom (but unquantified) pressures was observed for different pressure patterns in a previous study [19] for a 6 × 6 array of tactile sensors. This is the typical crosstalk effect in tactile sensors.

It has been theoretically shown that using an additional column of calibration resistors in the array (not sensors) eliminates the effects of crosstalk due to *RB_i_*, *θ_j_* and *Ib_j_* [30]. Furthermore, using an additional row of calibration resistors (not sensors), along with the previously added column, also eliminates the effect of finite gain in the OA.

This paper aims to, on an array of tactile sensors designed by the authors, show the operation of the circuit proposed in [30], and to assess the quality of the readings, in order to achieve a high-accuracy tactile sensor. Firstly, an array of resistors (not sensors) will be constructed to simulate the operation of the tactile sensor, and the errors that occur in the measurement of the values of the resistors will be evaluated. The range of resistor values used in this test will match the range of values provided by the tactile sensor to be used later. Finally, the taxels array will be connected to the electronic reading system and the obtained results will be displayed.

The structure of the paper is as follows: Section 2 shows the circuits that will be used to control the sensor array reading and solve the problem of crosstalk. Section 3 describes the electronic system implemented to test the proposed circuits. Section 4 shows the obtained experimental results and Section 5 analyses the consequences of using the equations detailed in Section 2. The final section summarizes the conclusions.

## 2. Electronic Reading Circuits

**Figure 3:**
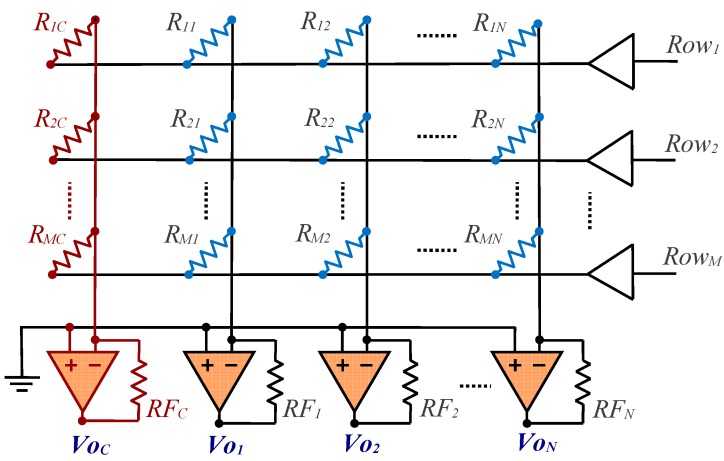
Circuit with the resistor array to be measured (in blue) along with the additional calibration column (in red) for Method I.

Type A circuit proposed in [30], as shown in Figure 3, will be used to eliminate crosstalk due to *RB_i_*, *θ_j_* and *Ib_j_*.

Compared to the Figure 1 circuit, the switches have been replaced with digital buffers controlled, in our implementation, directly by an FPGA. A column has been added to the array—*c*, the calibration column (shown in red in Figure 3)—which includes the same reading circuit as the other columns, as well as a calibration resistor with known value, *R_ic_*.

Continuing the analysis of this circuit that was described in [30], the value of *R_ij_* can be obtained from the following equation:

(2) $$R_{ij}=R_{ic}\frac{RF_{j}}{RF_{c}}\frac{Vo_{c}(0)-Vo_{c}(i)}{Vo_{j}(0)-Vo_{j}(i)}$$

Where *R_ic_*, *RF_j_* and *RF_c_* are resistors of known value and *Vo_c_*(0) is the output voltage of the OA of column *c* when all row selection buffers provide the ground voltage at the output. On the other hand, *Vo_c_*(*i*) is the output voltage of the OA of column *c* when row *i* has been selected. Likewise, *Vo_j_*(0) and *Vo_j_*(*i*) are the OA output voltages of column *j* for the same circumstances. We refer to this method of estimating the value of *R_ij_* as Method I.

In accordance with Equation (2), taking into account that a reading must be made when none of the rows are addressed, (*M* + 1)·(*N* + 1) readings are required to evaluate the *M*·*N* resistors of the sensor array for an array of *M*·*N* sensors. These readings are carried out in *M* + 1 reading cycles. This small increase in the number of readings and cycles required is the cost paid to avoid crosstalk due to *RB_i_*, *θ_j_* and *Ib_j_*.

**Figure 4:**
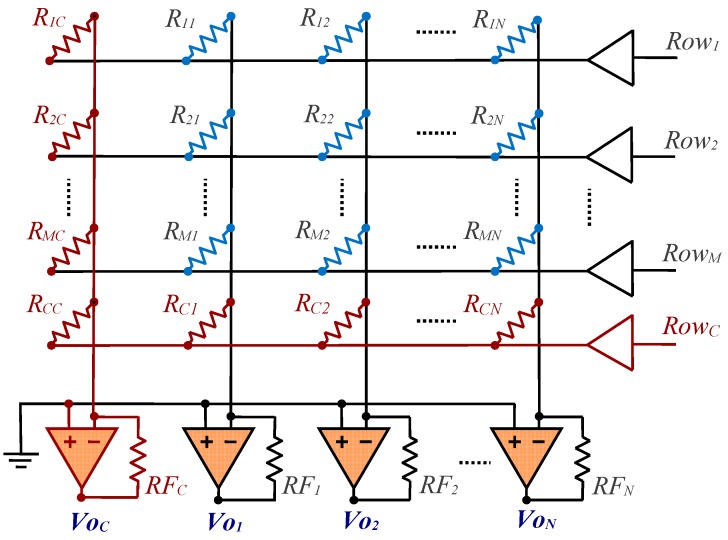
Circuit with the resistor array to be measured (in blue) along with the row and additional calibration column (in red) for Method II.

On the other hand, as described in [30], the circuit shown in Figure 4 will be used whenever it is necessary to avoid crosstalk due to a finite gain of the OA.

For this circuit, the value of *R_ij_* can be obtained from:

(3) $$R_{ij}=\frac{R_{ic}R_{cj}}{R_{cc}}\cdot \frac{Vo_{j}(0)-Vo_{j}(c)}{Vo_{j}(0)-Vo_{j}(i)}\cdot \frac{Vo_{c}(0)-Vo_{c}(i)}{Vo_{c}(0)-Vo_{c}(c)}$$

Where *R_ic_*, *R_cj_* and *R_cc_* are resistors of known value and the voltages are interpreted as in Equation (2). We refer to this method of estimating *R_ij_* using Equation (3) as Method II.

In this case, *N* additional resistors are required compared to the circuit in Figure 3, and (*M* + 2)·(*N* + 1) readings are needed to to evaluate the *M*·*N* resistors of the sensors. Consequently, the complete reading process consists of *M* + 2 cycles. There is an additional reading cycle compared to Method I.

## 3. Materials and Methods

**Figure 5:**
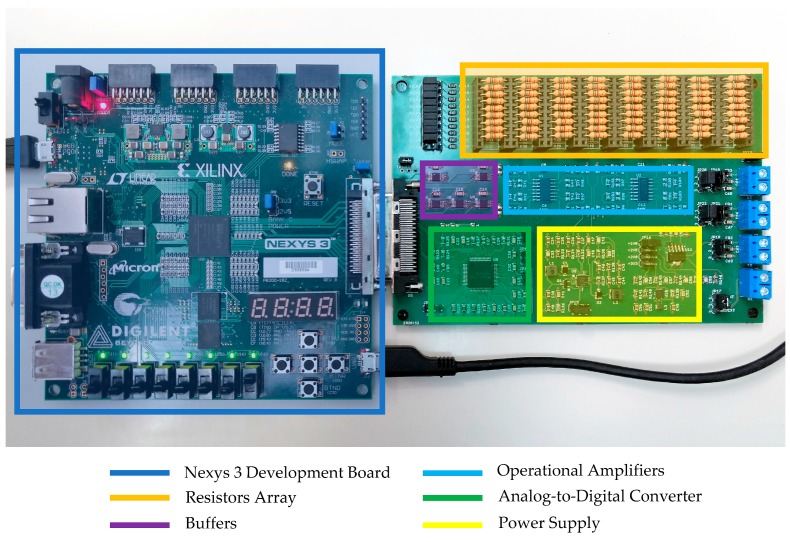
Electronic system used in the experiments presented.

**Figure 6:**
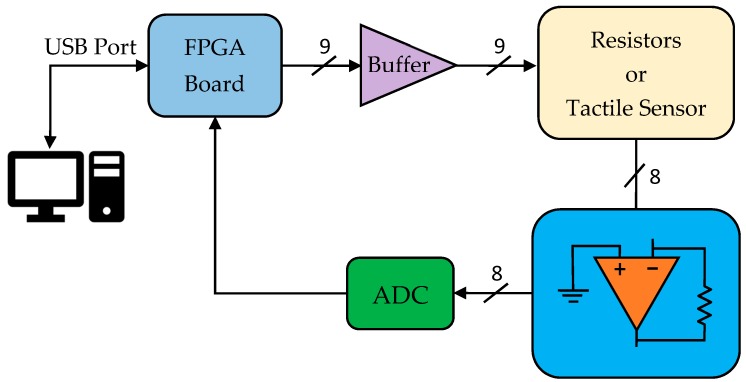
Block diagram of the whole system.

The proposed methods were tested with the electronics shown in Figure 5, and a block diagram of the whole system can be seen in Figure 6. The analog-to-digital converter (ADC) used was a model ADS8568 from Texas Instruments [31]. It is a 16-bit, 8-channel, simultaneous sampling, successive approximation register (SAR)-based ADC. Data are transferred to the FPGA via an SPI serial protocol. Row address was carried out using TS5A23159 analog switches from Texas Instruments [32]. The OAs used in this study were model OPA4188 from Texas Instruments [33]. Their main characteristics are: rail-to-rail input/output; input offset voltage: 25 μV (max); and low noise of 8.8 nV/√Hz.

Along with the circuit necessary for readings, a resistor array comprising 9 rows and 8 columns was constructed to carry out the experiments with resistors (not sensors) in the next section, allowing us to evaluate the quality of the readings provided by the system.

The resistor array consisted of eight rows and seven columns. In addition, one row and one column were used to measure the calibration resistors. Its values are indicated in the following section for each of the experiments carried out.

A Nexys 3 Spartan-6 FPGA development board [34] was used to send the acquired data to a host computer. Since the purpose of the tests was only to determinate the quality of the measurements, the sampling frequency of the array during the tests was set as 50 frames per second (fps). Higher sampling rates can be achieved by optimising interface electronics, as the ADC has a maximum data rate per channel of 480 kSPS.

**Figure 7:**
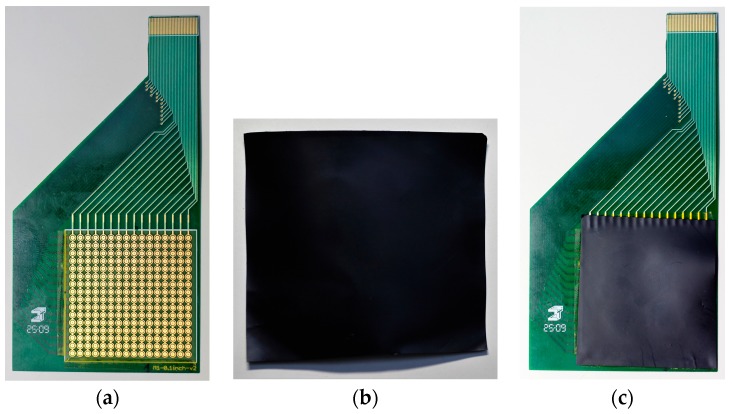
(**a**) Array of electrodes; (**b**) Piezoresistive sheet; (**c**) Full piezoresistive tactile sensor.

The resistor array (except the calibration resistors) was then replaced with a tactile sensor designed by the authors. The tactile sensor was made by placing a continuous electroactive sheet on an array of electrodes. This array had 16 × 16 pairs of electrodes on a flexible printed circuit board (FR4 0.2 mm width). Each pair of electrodes had an inner circular electrode (1.5 mm^2^ area) and a surrounding ring-shaped electrode (1.55 mm^2^ area), with a gap between electrodes of 0.1 mm. The electrodes were made of copper with a chemical Ag surface finish. The resulting thickness of the conductive layer was 35 µm. The distance between the centers of two taxtels along both vertical and horizontal axis was 2.54 mm. The active area of the tactile sensor was 16.52 cm^2^. The inner electrodes were connected to each, other forming rows, while the outer electrodes were connected to form columns. The structure appears in Figure 7a. Subsequently, the electrodes were covered with a continuous electroactive material using a piezoresistive sheet of capLINQ (code MVCF-40012BT50KS/2A). This sheet had a width of 0.1 mm and a surface resistance of 50,000 Ohm/cm^2^. The piezoresistive sheet appears in Figure 7b, while Figure 7c shows the complete sensor. Built in this way, the tactile sensor was able to cover the resistance range studied in the paper, as shown in Table 1 and Table 2.

**Figure 8:**
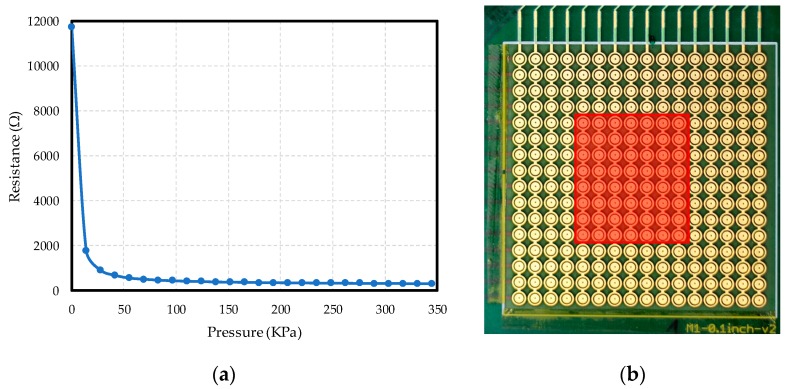
(**a**) Average calibration curve for sensors in the array; (**b**) Active area of the tactile sensor used.

An average calibration curve of all the taxels is shown in Figure 8a. The tactile sensor behaves such that, the higher the pressure, the lower the output resistance between the inner and outer electrodes.

Although the tactile sensor comprised 16 × 16 taxels, only 8 rows and 7 columns of taxels were connected, in order to ensure compatibility with the resistor array used in evaluating the quality of the reading electronics. Meanwhile, column 8 was used for Method I and row 9 and column 8 for Method II (they are used for calibration purposes for the resistor array). The active area of the tactile sensor was the subarray of taxels from rows five to twelve, and from columns five to eleven, as can be seen in the red area in Figure 8b.

## 4. Results

Two experiments with discrete resistors, under two different conditions, were implemented to analyse the performance of both the circuits proposed in Equation (2), Method I (M.I), and Equation (3), Method II (M.II). The performance of these circuits was compared to Equation (1), Classical Method (CM).

### 4.1. Experiment 1

Resistors in the 270–10,000 Ω range were measured using 1400 Ω as the nominal value for the calibration resistors. The rest of the resistors of the array took the mid-range value 5600 Ω, including the row and column of the resistor under test. The results are shown in Table 1.

The results of $\bar{R}$ and σ were obtained after carrying out 500 measurements, while the maximum relative error column in Table 1 shows the worst case out of the 500 measurements. The same procedure was used in the other experiment described in this section.

### 4.2. Experiment 2

Resistors in the 270–10,000 Ω range were measured using 1400 Ω as the nominal value for the calibration resistors. The other resistors in the array took the mid-range value 5600 Ω, except for the row and column of the resistor being tested, which had the minimum value (270 Ω), this being the worst situation in terms of crosstalk in the array when evaluating the value of a resistor. The results are shown in Table 2.

**Figure 9:**
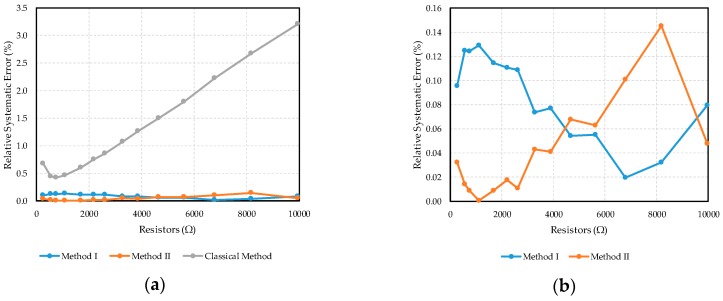

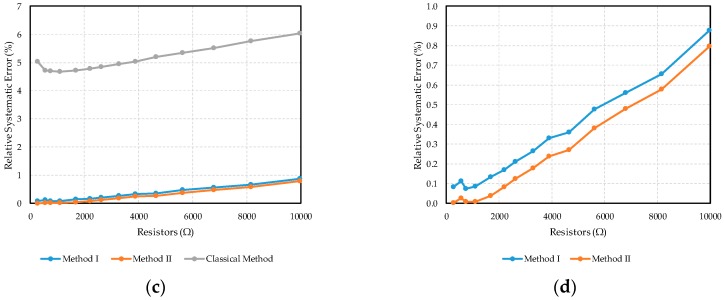
(**a**) Relative systematic error for Experiment 1; (**b**) Further detail of Method I and II relative systematic error for Experiment 1; (**c**) Relative systematic error for Experiment 2; (**d**) Further detail of *Method I and II* relative systematic error for Experiment 2.

The relative systematic error obtained for each of the methods in both experiments is given by the equation $\left|R-\bar{R}\right|/R$. These errors are shown in Figure 9.

### 4.3. Experiment 3

**Figure 10:**
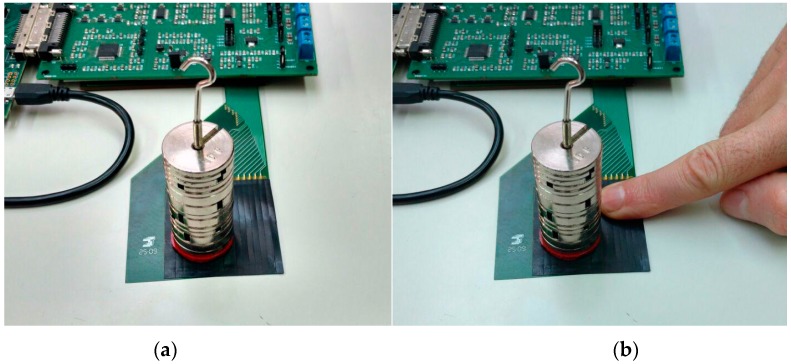
(**a**) Tactile sensor pressed with a weight of 200 g; (**b**) Tactile sensor pressed in two different areas with a weight of 200 g and a finger.

A third experiment was carried out with the piezoresistive tactile sensor shown in Figure 7. The aim was to show the reduction of the influence of the crosstalk in the tactile sensor. First, a tactile image was obtained with a weight of 200 g on the sensor (Figure 10a). Then, the sensor was pressed with a finger on a different location, and a second image was captured (Figure 10b). We compared the most pressed taxel in both images to show the effect of pressing in more than one area of the tactile sensor.

The value of the resistance of the taxel calculated with the Method I in the first case (Figure 10a) was 5563 Ω, and its value when another area of the sensor was pressed with a finger (Figure 10b) was 5498 Ω. Therefore, the relative error due to the crosstalk was 1.18%.

As a final experiment, a video demonstration (included in the additional materials) has been included to show the behavior of the system with the tactile sensor connected. This shows how information can be obtained from the tactile sensor (in this case, simultaneous movement of several fingers at a time is shown) using Equation (2) to determine sensor resistance.

## 5. Discussion

As shown in the graphs in Figure 9, Methods I and II both improve on the results obtained by using Equation (1) By almost one order of magnitude. In general, the errors arising from the Classic Calculation Method increase with the value of the resistor, while for Methods I and II the errors remain approximately independent of the resistance value to be measured.

This increment of the error for the Classic Calculation Method is due to the fact that if the resistor being tested increases its value, the total current through it decreases. Thus, the current component due to crosstalk becomes more significant.

Moreover, errors measured in Experiments 1 and 2 corresponding to Method I and II are quite similar as can be seen in Figure 9b,d. This shows that the resistance value of the other resistors has a negligible effect on the resistor under test.

Although, theoretically, Equation (3) should provide better results than Equation (2), the fact that a greater number of readings are required in Method II (resulting in a higher standard deviation of the results, as shown in Table 1 and Table 2) and the fact that the OAs used in the circuit have a very high gain (meaning the influence of this parameter is small) explain why this benefit is not observed in Figure 9b. Experiment 2 does show a slight improvement for Method II, although the difference may not justify the extra hardware and readings involved. Also, a slight error dependence on the value of the resistor is evident in the graphs of Experiment 2. This can be explained by the relative error increase in the analog-to-digital conversion for small voltage values which are obtained at the OA outputs for the high resistance values to be measured.

Considering that Method I presents similar results to those obtained by Method II, the simpler first method was used to evaluate the effect of pressing in a second location on the tactile sensor in Experiment 3. The number of taxels that are pressed in this second location is high, therefore the influence of crosstalk should be significant. However, the resistance of the reference taxel under the first object varies by only 1.18%.

Moreover, the video included in the additional materials does not show any type of “phantom” pressure in the array while several fingers are moving, which indicates the suppression of crosstalk.

## 6. Conclusions

Two methods for eliminating crosstalk in a resistive sensor array have been evaluated. Both methods showed better results than the equation traditionally used to evaluate the values of individual resistors. The comparison was carried out by constructing an electronic system that enabled both an array of discrete resistors (to verify the performances of each reading method) and a tactile sensor designed by the authors to be used.

Furthermore, it has been shown that, although Method II should show better results, in practice it provides similar results to those obtained with Method I if the circuit OAs are chosen properly.

## Figures and Tables

**Table 1:** Accuracy data for *Rij* estimation with a value of 5.6 KΩ for the row and column of the resistor under test.

Resistor (Ω)	$\bar{R}(\Omega )$			σ (Ω)			$\left|R-\bar{R}\right|/R(\%)$			Max. Rel. Error (%)		
	CM	M.I	M.II	CM	M.I	M.II	CM	M.I	M.II	CM	M.I	M.II
267.8	269.61	267.54	267.89	0.06	0.12	0.26	0.67	0.096	0.0325	0.73	0.31	0.31
556.2	558.69	555.50	556.12	0.20	0.22	0.55	0.45	0.125	0.0141	0.53	0.25	0.27
747.5	750.67	746.57	747.43	0.33	0.35	0.71	0.42	0.125	0.0090	0.53	0.26	0.26
1097.3	1102.42	1095.88	1097.30	0.65	0.43	1.11	0.47	0.129	0.0004	0.62	0.25	0.25
1685	1695.13	1683.07	1685.15	1.60	0.89	1.59	0.60	0.115	0.0088	0.80	0.33	0.25
2198.4	2214.83	2195.96	2198.79	2.61	1.24	2.12	0.75	0.111	0.0179	1.00	0.36	0.24
2616.1	2638.47	2613.25	2616.38	3.99	1.76	2.63	0.86	0.109	0.0108	1.32	0.36	0.31
3282.6	3317.65	3280.18	3284.01	5.97	2.74	3.47	1.07	0.074	0.0431	1.48	0.40	0.33
3883.2	3932.15	3880.20	3884.80	8.28	3.81	4.55	1.26	0.077	0.0412	1.73	0.39	0.44
4656.5	4726.44	4653.98	4659.65	12.05	5.83	6.20	1.50	0.054	0.0677	2.10	0.59	0.50
5621.4	5722.61	5618.29	5624.93	17.47	8.58	9.03	1.80	0.055	0.0628	2.50	1.04	1.21
6789.6	6940.56	6788.28	6796.44	26.86	13.42	13.49	2.22	0.019	0.1008	3.06	0.96	1.20
8170.5	8388.56	8173.12	8182.39	39.12	18.20	17.92	2.67	0.032	0.1456	4.33	1.21	1.41
9963.2	10,282.45	9955.25	9967.98	59.58	23.95	25.59	3.20	0.080	0.0480	4.32	0.92	0.93

**Table 2:** Accuracy data for *Rij* estimation with a value of 270 Ω for the row and column of the resistor under test.

Resistor (Ω)	$\bar{R}(\Omega )$			σ (Ω)			$\left|R-\bar{R}\right|/R(\%)$			Max. Rel. Error (%)		
	CM	M.I	M.II	CM	M.I	M.II	CM	M.I	M.II	CM	M.I	M.II
267.8	281.26	267.57	267.79	0.07	0.13	0.27	5.024	0.084	0.003	5.09	0.23	0.28
556.2	582.40	555.57	556.05	0.23	0.24	0.56	4.710	0.112	0.026	4.82	0.24	0.24
747.5	782.62	746.96	747.56	0.35	0.34	0.72	4.699	0.073	0.007	4.82	0.21	0.29
1097.3	1148.69	1096.37	1097.19	0.76	0.49	1.11	4.683	0.085	0.010	4.84	0.23	0.25
1685	1764.66	1682.75	1684.35	1.63	0.96	1.56	4.728	0.133	0.039	4.96	0.33	0.26
2198.4	2303.55	2194.70	2196.60	2.87	1.48	2.22	4.783	0.169	0.082	5.05	0.42	0.41
2616.1	2742.63	2610.56	2612.82	4.00	2.06	2.74	4.837	0.212	0.125	5.18	0.50	0.52
3282.6	3445.09	3273.91	3276.74	6.24	3.06	3.56	4.950	0.265	0.178	5.33	0.58	0.47
3883.2	4078.77	3870.33	3873.94	9.06	5.08	5.51	5.036	0.332	0.239	5.86	0.90	0.70
4656.5	4898.48	4639.66	4643.91	13.15	6.85	7.41	5.197	0.362	0.270	5.72	1.00	0.98
5621.4	5921.96	5594.59	5599.92	19.02	10.84	11.32	5.347	0.477	0.382	6.07	1.34	1.13
6789.6	7164.40	6751.60	6756.99	29.33	12.53	14.07	5.520	0.560	0.480	6.54	1.15	1.13
8170.5	8641.64	8116.76	8123.23	40.25	24.32	24.42	5.766	0.658	0.579	6.77	1.94	1.82
9963.2	10,564.45	9875.72	9883.93	60.05	30.64	30.59	6.035	0.878	0.796	7.31	2.44	2.32

## Supplementary Materials

The following are available online at http://www.mdpi.com/1424-8220/17/11/2513/s1, Video S1: High-precision Readout Electronics for Piezoresistive Tactile Sensors.mp4.
